# The Development and Validation of the Partial Denture Experience Questionnaire (P‐DEQ): Reliability and Validity

**DOI:** 10.1111/ger.70063

**Published:** 2026-03-05

**Authors:** Barry J. Gibson, Nicolas Martin, Bilal El‐Dhuwaib, Gerry McKenna, Sandra Clifford, Alastair Lomax, Sarah R. Baker

**Affiliations:** ^1^ School of Clinical Dentistry University of Sheffield Sheffield UK; ^2^ School of Medicine, Dentistry & Biomedical Sciences The Queen's University of Belfast Belfast UK; ^3^ Haleon Weybridge UK; ^4^ Lincoln Institute for Rural and Coastal Health Lincoln University Lincoln UK

## Abstract

**Introduction:**

This paper describes the development and preliminary validation of the Partial Denture Experience Questionnaire (P‐DEQ), a new, condition‐specific instrument designed to measure the multifaceted impacts of living with removable dentures.

**Methods:**

The P‐DEQ was developed using a multi‐phase, mixed‐methods design. Item generation was informed by qualitative interviews (*n* = 20) with denture wearers in the UK and guided by the World Health Organisation's International Classification of Functioning, Disability and Health (ICF) framework. A 34‐item scale, with five core sub‐scales (Body Function, Emotional Function, Daily Abilities, Social Impacts and Participation Restrictions) and a separate ‘My Denture’ sub‐scale, was tested in a cross‐sectional study with online panels from the UK (n‐224) and US (n‐224). Reliability, item‐total correlations, item impacts and sub‐scale correlations were assessed.

**Results:**

The P‐DEQ sub‐scales demonstrated good to excellent reliability (Cronbach's α = 0.71–0.88) across both samples. Sub‐scale‐to‐total score correlations were high and significant, with the Emotional Function (*r* = 0.92) and Social Participation (*r* = 0.92) sub‐scales showing particularly strong relationships in the UK sample. Most item‐total correlations exceeded the 0.4 threshold, supporting the instrument's underlying coherence. Item impact scores varied widely; items concerning psychosocial worries (e.g., the denture breaking) and functional limitations (e.g., avoiding certain foods) registered the highest impact. In contrast, items reflecting potential benefits of the denture, such as improved appearance, had lower impact scores. Furthermore, the ‘My Denture’ sub‐scale, measuring personal appraisal of the prosthesis, was significantly correlated with the total P‐DEQ score (*r* = −0.50 UK; *r* = −0.55 US), indicating that a more favourable personal evaluation of a denture was associated with fewer negative impacts.

**Conclusions:**

The P‐DEQ demonstrates promising reliability and content validity for assessing the complex experience of living with a removable denture. The instrument is sensitive to the nuanced, often ambivalent, emotional states of wearers. Whilst this initial validation is based on cross‐sectional data, longitudinal testing is required for item reduction and to fully establish the P‐DEQ as a robust measure that can detect change over time.

## 1 Introduction

Tooth loss remains a significant public health issue globally, affecting nearly 4 billion people, particularly older adults, worldwide [1, 2]. While advancements in preventive dentistry have led to a decline in complete edentulism, severe tooth loss (defined as having fewer than 9 remaining permanent teeth) remains significantly high in some regions (Brazil, Turkey, Iran, Mexico and New Zealand) [2]. In the United Kingdom, data from the 2021 Adult Oral Health Survey (AOHS) for England indicated that 5% of adults aged 16 and over were edentate, and 11% reported wearing a denture replacing one or more teeth [3]. Earlier UK‐wide data from the 2009 Adult Dental Health Survey (ADHS) found 6% of adults were edentate and used full dentures, while a further 13% used partial dentures, totalling almost 20% using some form of denture [4]. Denture use markedly increases with age; the 2021 survey showed prevalence rising from 1% in 16–24‐year‐olds to 39% in those aged 75 and over. Furthermore, a clear socio‐economic gradient exists, with higher prevalence observed in lower‐income households and more deprived neighbourhoods. Denture wearing is therefore closely related to social inequalities with complete and partial dentures still amongst the most common forms of treatment for tooth loss [3, 4]. These treatments are aimed at restoring oral function, aesthetics, and have the potential to improve oral health related quality of life (OHRQoL) [5, 6].

Clinical research has traditionally focused on the functional outcomes of denture provision, measured through masticatory efficiency, stability, retention and the longevity of the prostheses [6, 7]. However, the impact of living with dentures extends far beyond these clinical indicators with patients' reporting significant impacts on their daily experience, including impacts on their social interactions, emotional well‐being, self‐esteem and daily activities [8, 9]. Qualitative studies have consistently highlighted the significant psychosocial burden that can accompany denture use, including embarrassment during eating or speaking, fear of denture displacement, social avoidance and altered self‐perception [9, 10, 11]. These aspects are crucial for understanding the success of prosthetic rehabilitation from the patient's perspective.

Various instruments have been developed to assess Oral Health Related Quality of Life (OHRQoL) with generic OHRQoL measures, such as the Oral Health Impact Profile (OHIP) and its shortened versions (e.g., OHIP‐14, OHIP‐20sp), widely used and validated [7, 8, 9]. While valuable for capturing broad oral health impacts, these generic tools can lack the specificity required to fully encompass the unique experience associated with wearing removable partial dentures. Research has demonstrated that in treatment with Removable Partial Dentures (RPDs), OHIP‐14 scores improve in the short term (within 6 months) but tend to show little or no change at 1 year [12]. This is potentially because the most significant changes happen in the first 3 months when the RPD replaces missing teeth. It could also be because participants adjust their expectations and scores return to their baseline in the light of these changing expectations over a longer period, a phenomenon called ‘response shift’ [10, 11]. It is also possible that there are floor and ceiling effects with RPDs failing to deliver on patient expectations [6]. It may be, therefore, that there are subtle changes happening that cannot be picked up by generic OHRQoL instruments.

Instruments have been developed that focus on specific populations or aspects of prosthetic experience, for example, the Quality of Life with Implant‐Prostheses (QoLIP‐10) questionnaire for implant patients assesses chewing function [13, 14, 15], or those focusing specifically on the emotional and social issues related to eating with dentures (ESIRE) [16]. Furthermore, the use of psychological tools like the Depression, Anxiety and Stress Scale (DASS‐21) has revealed that people who have a diagnosis of depression (current or lifetime) along with lifetime diagnosed anxiety have been found to be significantly more likely to have at least one tooth removed compared to those without these disorders. These relationships were found to hold after adjusting for confounders [17]. Many of these scales provide a partial insight into the specific challenges associated with living with a removable denture [18]. With these in mind the OHIP‐EDENT and prosthetic quality of life (PQL) questionnaires were developed [18].

The OHIP‐EDENT was developed in response to the fact that the shortened form of the OHIP, the OHIP‐14, demonstrated that there was a high proportion of scores from *edentulous* patients that were ‘0’ indicating that there was no impact for a large number of the statements [19]. Allen and Locker [19] argued that this may have been because of the use of factor analysis in the development of the OHIP‐14 which can remove items that are important to different groups of patients. In response to this the OHIP‐EDENT was derived from two samples of edentulous participants who had completed the OHIP‐49 using the ‘item impact approach’ first reported by Juniper et al. [20]. The OHIP was itself derived from statements about the impact of oral health taken from interviews with 64 dental patients in 1992. These statements were subsequently refined and selected using Thurstone's method of paired comparisons [7]. These items were sorted into the seven domains of Locker's model of oral health [21]. The full range of items that form the OHIP‐EDENT along with Locker's model of oral health are reproduced in online Appendix S1.

The PQL questionnaire was developed by a panel of experts who read a series of interviews with 44 patients discussing how their ‘oral health related well‐being was affected by their removable prothesis’ [18]. The resulting questionnaire was pilot tested with 36 individuals to check face and content validity. The response set for each question is very different (see online Appendix S1). This makes it hard to be certain that when combined the underlying scale is assessing the same or even similar underlying constructs. Items assess perceived need for treatment, complaints about the mouth, self‐reported oral satisfaction and so on. These constructs are not directly related to any established model of oral health [18, 22]. Such models are essential to ensure that indicators systematically cover all aspects of the underlying constructs associated with oral related quality of life [23, 24, 25].

The OHIP was developed over 30 years ago now. It was based on Locker's model of oral health [21] which itself was developed from a modified form of the World Health Organization's International Classification of Impairments Disability and Handicap (ICIDH) [26], informed by earlier ideas about illness [27, 28]. Locker's ideas about oral health were also informed by debates around how to link clinical indicators to oral health and quality of life outcomes [29, 30]. These conceptualisations are central in the move away from services being focused on a purely biomedical model of disease towards a more person‐centred approach. Many of the underlying constructs are, however, subject to debate, with various modifications being suggested in the last 20 or so years [24, 31, 32].

**FIGURE 1:**
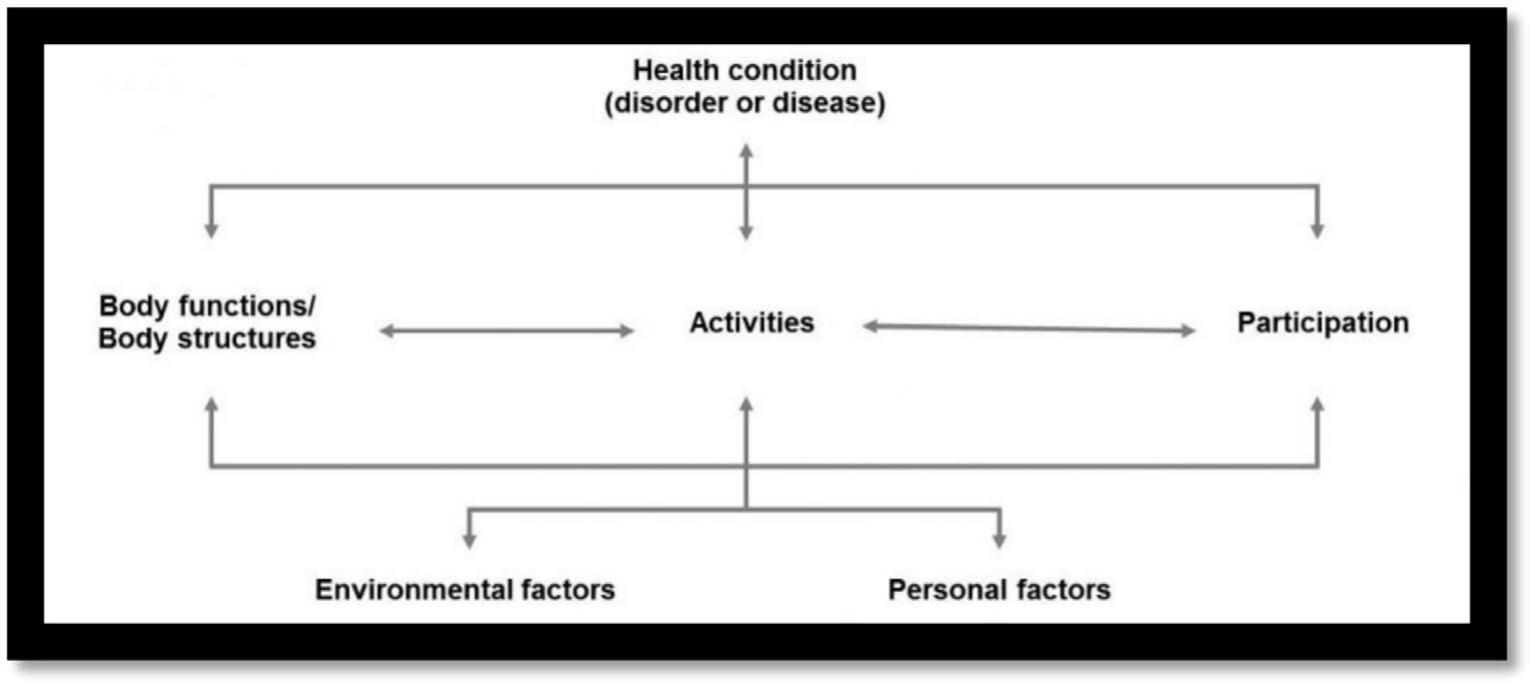
The ICF model. (*Source:* WHO 2001)

The ICIDH has been challenged for being too individualised and placing the burden of disease on the shoulders of the disabled [33, 34, 35, 36]. These criticisms eventually led to the development of a new model called the ‘International Classification of Functioning Disability and Health’ (ICF) (Figure 1). This model is critical in disability rights activism because its development included the views of disabled people themselves. The model seeks to conceptualise everyday functioning and disability as outcomes of *the interaction between health conditions (disorder or disease), body functions and structures, activities and participation alongside environmental and personal factors* [31].

The new framework marked an important development in the understanding of disability. Disability is no longer defined in terms of the impact of body functions and limitations on individuals; it is now seen as the product of the process through which bodily functions and abilities *interact with the environment*. As Figure 1 seeks to illustrate, it is the *interaction* between these factors that leads to social exclusion and disability [37].

Added to this important development are several additional factors. Subsequent to Locker's Model being developed, authors working in the sub‐field of oral and dental rehabilitation have argued for a better appreciation of the positive aspects of oral health [24, 38, 39]. This involves tackling the thorny issue of positively worded items [38] whilst considering how the benefits of oral rehabilitation might best be captured. It means better understanding that whilst individuals may be experiencing negative aspects to their oral health, they might also be experiencing relative health alongside these impacts, a phenomenon termed the paradox of health [40, 41, 42]. A recent comprehensive review of OHRQoL measures demonstrates that almost all measures currently in use refer to Locker's conceptual model [21, 22]. There is no record of any measures being developed with these new ideas about illness in mind. Although new directions in this field are now quickly emerging [43].

The field of oral health has expanded considerably since the early 1990's [22]. Increasing attention has been given to the impact and experience of multiple oral conditions. There are now a plethora of studies looking at the various impacts of different conditions such as Sjögren's Syndrome and dry mouth [44, 45, 46], dentine hypersensitivity [47, 48], tooth loss [49, 50] and Temporomandibular Disorder (TMD) [51] to name just a few. Many of these developments have resulted from a desire to be better able to detect change in oral conditions. Just as the OHIP‐14 could not detect impacts in patients who are edentate, the OHIP‐14 has been shown to be insensitive to change in many other conditions [52]. Guyatt and Tugwell [53] argued that the purpose of a quality‐of‐life measure might be evaluative, or to detect change; it was with the latter in mind that the OHIP‐Edent was developed [19].

There remains no comprehensive patient‐centred questionnaire designed explicitly to measure the multifaceted aspects of OHRQoL impacts experienced by people who have lost teeth and who are living with *removable partial dentures* (RPDs). Such an instrument should reflect changes in the conceptualisation of disability (see Figure 1) and seek to integrate functional, psychological, emotional and social dimensions. Guyatt and Tugwell [53] are clear that such measures should be derived directly from patient experiences, and their purposes should be clearly stated. A fully validated, condition specific tool would enable the measurement of change and so enable the comparison of outcomes across different oral health related interventions [53, 54].

The aim of this study is to describe the development and validation of a new condition specific instrument, the Partial Denture Experience Questionnaire (P‐DEQ). The P‐DEQ is designed to comprehensively assess OHRQoL impacts associated with wearing removable partial dentures. This study details the methodology employed in the questionnaire's design, including item generation and reports on the cross‐sectional evaluation of its psychometric properties in two populations from the UK and the US.

## 2 Materials and Methods

### 2.1 Study Design

**FIGURE 2:**
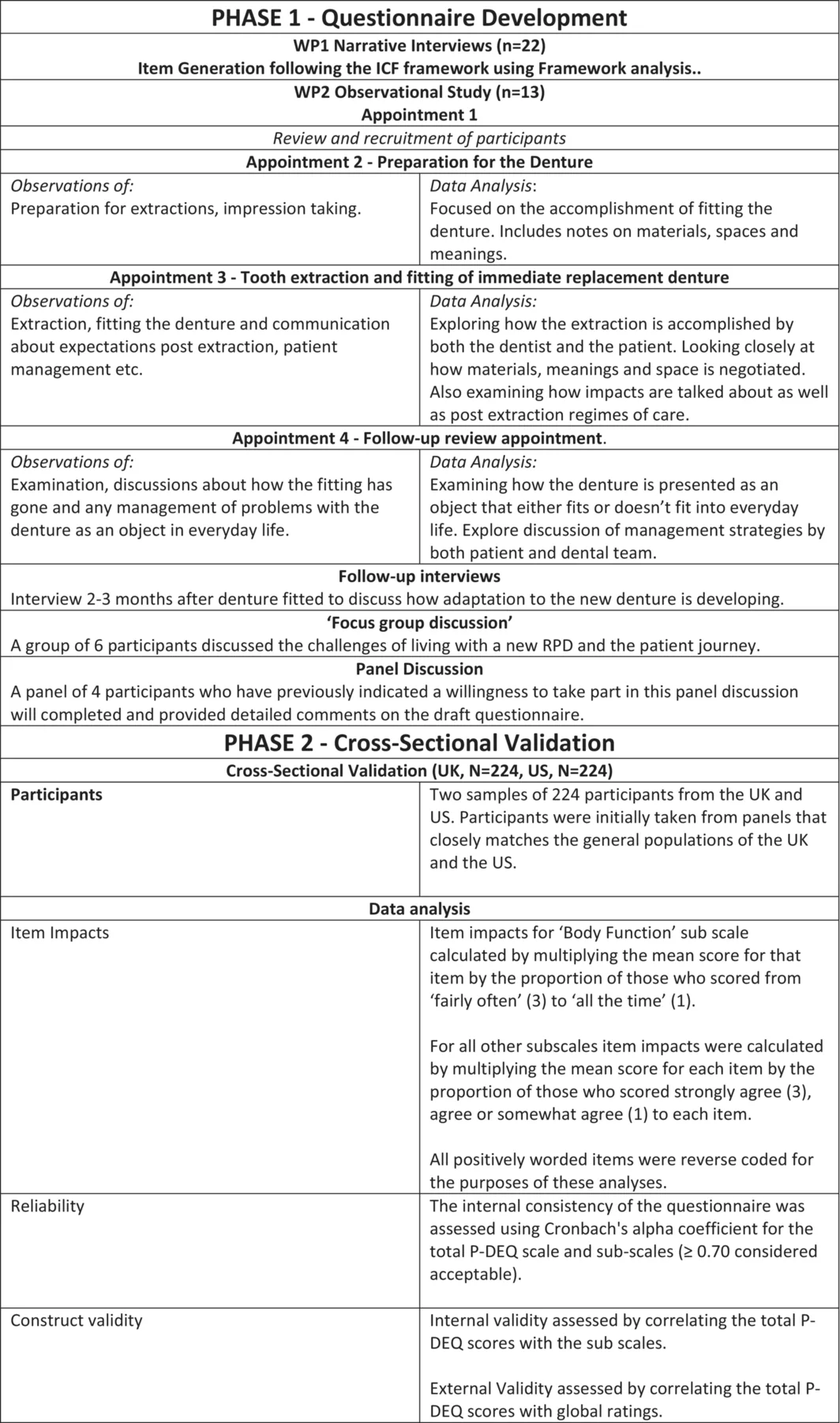
Study Flow Diagram.

This study employed a multi‐phase, mixed methods design to develop and validate the Partial Denture Experience Questionnaire (P‐DEQ) with the overall approach summarised in Figure 2. Phase 1 involved qualitative methods for item generation and content development, including clinical observations, semi‐structured interviews, expert consultation and a focus group. Phase 2 involved a cross‐sectional study in two populations of partial denture wearers recruited by Ipsos from the UK and the US. The goal of this research programme is to develop a measure that reflects the priorities of patients and clinicians and follows that developed by Guyatt and colleagues [20, 53, 54].

### 2.2 Ethics Statement

Ethical approval for Phase 1 was provided by the NHS (IRAS number 312493) and the University of Sheffield Research ethics committee (application number 047005). The Second phase was approved by Ipsos and the University of Sheffield (application number 068385).

For Phase 1 all participants provided written informed consent, after receiving a detailed patient information sheet 1 week prior to talking about their participation with the research team. Before taking part, researchers explained the study's purpose, procedures, confidentiality and their right to withdraw at any time without penalty. Participant anonymity and data confidentiality were maintained throughout the study.

### 2.3 PHASE 1: Questionnaire Development

#### 2.3.1 Theoretical Model

**FIGURE 3:**
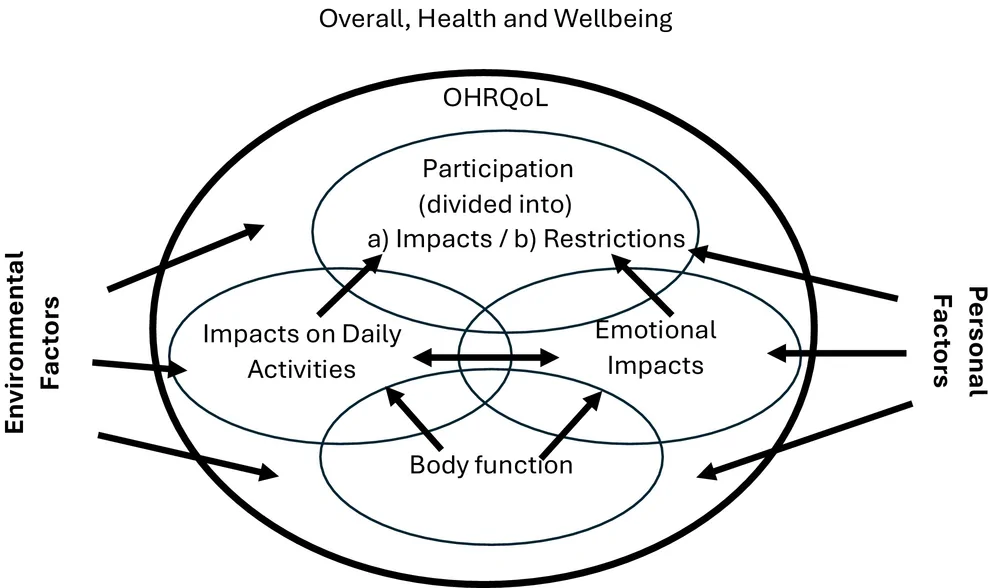
The ICF adapted for living with a denture.

The theoretical model used to build the P‐DEQ measure was the International Classification of Functioning Disability and Health (ICF) from the World Health Organisation (see Figure 1). This model was chosen because of its focus on the interaction between conditions, the person and their environment and the focus on participation. It was also chosen because it is suited to capture the positive effects of wearing a denture [24, 39]. The goal behind the model is to provide a common language and framework with which to talk about health and disability. Although the arrows go in multiple directions, it was always made clear that researchers should hypothesise how a condition might be operationalised. It was therefore decided to follow the work of Ravenek et al. [55] in their clarification of the ICF. They hypothesised the adopted model as presented in Figure 3.

The P‐DEQ was designed to measure everything within the inner circle but also to include items that were deemed important to participants, for example, the emotional impact of tooth loss has long been established as an important aspect of the experience [49, 56, 57]. At this stage the overall questionnaire had 62 items, of which 34 comprised the P‐DEQ impact scale. The five sub‐scales of the P‐DEQ are Body Function, Emotional Impacts, Impacts on Daily Activities, Participation, divided into (a) impacts and (b) restrictions. Alongside these, there are questions on how people appraise their denture (MYDenture sub scale), together with global health ratings related to oral health, general health, happiness with their denture and overall quality of life.

#### 2.3.2 Item Generation and Domain Identification

The initial item pool for the P‐DEQ was generated through several complementary approaches. We completed narrative interviews with (*n* = 20) participants purposively recruited from the public and the NHS through advertising online through websites such as the University of the Third Age, and forums such as Reddit. The intention was to gather a wide range of perspectives and experiences of living with a denture. The goal was to recruit participants with a range of ages, socio‐economic backgrounds and an equal gender split.

Participants included those who were aged 18 and above, and who had a partial denture fitted in the past 5 years. The interviews started by asking participants to describe the story of their denture (‘could you tell me about your denture?’), before moving on to the history of the denture and tooth loss (pre‐extraction, post extraction, pre denture fitting, post denture fitting) and changes that had occurred over time. The goal was to cover all aspects of the participant's experiences with their denture. These interviews were analysed using Framework Analysis [58, 59] with the ICF acting as the framework (Figure 1) using NVivo software Version 1.7.1 [60].

This analysis moved through a series of stages where statements from participants about their everyday experience of living with a removable partial denture were taken and directly fitted into the categories associated with the domains of the ICF. These included Personal Factors, Body Function, Emotional Impacts, Impacts on Daily Activities, Participation (a) Impacts and (b) Restrictions. Items were, wherever possible, framed directly from participants' statements so, for example, one participant stated that ‘My denture is part of me’. This then became part of the personal factors construct ‘MYDenture’ as the statement ‘My denture is a part of me’. Or in another example Bobby stated ‘The taste, you lose a lot of taste with a plastic one, the one with the hood on sort of thing. You lose the sense of taste. When you were drinking something, it was like washing the top and trying to come down. Sometimes you were like nearly losing it. It like separated from your mouth’. This became Body Function item ‘My denture affects my sense of taste for some foods or drinks’.

##### 2.3.2.1 Clinician Input

To ensure good content validity three members of the research team were experienced clinicians working in primary and secondary care all of whom provide dentures on a regular basis. All were involved in discussions about the initial item pool and the clinical observations; they actively suggested the inclusion of important sub‐scales and the rewording of many items.

#### 2.3.3 Initial Item Pool and Reduction

The themes and statements derived from the interviews and clinician input were synthesised to create an initial pool of 61 items; these were very loosely organised into 8 domains. These were then discussed at several meetings of the whole team and rearranged/reorganised into the appropriate themes. The resulting pool was gradually reduced to form the overall questionnaire that encompassed 7 domains including personal factors, environmental factors, body function, emotional impacts, impacts on daily activities, participation (a) impacts and (b) restrictions. This pool was reviewed by the research team (including a specialist prosthodontist, a restorative dentist and a general dental practitioner, a health psychologist, geographer and sociologist) for relevance, clarity, redundancy and overlap. Items that were ambiguous, repetitive or deemed less relevant to the core constructs of the denture experience were removed or rephrased.

#### 2.3.4 Panel Testing

To examine the face validity of the first draft of the P‐DEQ, a panel test was conducted with four individuals who had taken part in the interviews. Due to the relatively low number of volunteers for the study and differing ethical agreements between those recruited through the university and the NHS, all of the panel interviews were conducted with female participants. This group had an age range of 22–62 years.

### 2.4 PHASE 2: Cross‐Sectional Validation

#### 2.4.1 Participants

The initial sample was recruited from two Ipsos panels taken from the UK and the US general populations using Ipsos's ‘PanelOne’ (online panel of respondents). PanelOne involves daily mailouts to potential respondents with a survey digest of potential studies for them to consider taking part in. They then access the survey through a link in the digest, and PanelOne redirects potential participants to studies that match their profiles. Recruitment aimed to match gender and other demographic variables wherever possible in both samples. PanelOne is contained in an online proprietary platform that closely mirrors the general population. In this instance Ipsos also used external panels to widen the reach of sampling, especially in getting enough subjects given the niche nature of the population.

#### 2.4.2 Data Analysis

A cross‐sectional validation was carried out which included item impacts, construct validity, sub‐scale analysis, internal reliability as follows:

#### 2.4.3 Item Impacts

Item impacts for the ‘Body Function’ sub‐scale were calculated by multiplying the mean score for that item by the proportion of those who scored from ‘fairly often’ (3) to ‘all the time’ (1). All other sub‐scale item impacts (Emotional Function, Daily Abilities, Social impacts and Participation restrictions) were calculated by multiplying the mean score for each item by the proportion of those who scored strongly agree (3), agree or somewhat agree (1) to each item. Item impacts for the MYDenture sub‐scale were calculated by multiplying the mean score for each item by the proportion of those who scored strongly agree, agree or somewhat agree to each item. All positively worded items were reverse scored for consistency.

#### 2.4.4 Reliability

The internal consistency of the questionnaire was assessed using Cronbach's alpha coefficient for the total P‐DEQ scale and sub‐scales (*α* ≥ 0.70 is considered acceptable).

#### 2.4.5 Construct Validity

Construct validity was assessed by correlating the total P‐DEQ scores with the sub scales (internal validity) and global ratings (external validity). Although it is often stated that confirmatory factor analysis is a gold standard method for reducing items [22] this is not the only approach. Guyatt and colleagues [53, 54] argue for person centred approach that involves prioritising patients and clinicians perspectives over statistical methods. This approach, termed the ‘item impact approach’, was employed by Slade and Spencer [7] in the original development of the OHIP. It was only when they needed to reduce items into a shorter form that factor analysis was used [8]. We follow this approach here. The data were analysed using SPSS 25.0.1.1 [61].

## 3 Results

The items for this questionnaire were derived from a sample of 20 individuals (11 males and 9 females) aged between 22 and 86. Participants came from a broad range of social backgrounds including students, unemployed, retired, technical and professional backgrounds (See Online Appendix S1, Table S4). Panel testing recommended moving from a 5‐point Likert scale for the impact items to a seven‐point Likert scale and to clearly add the numbers that would be used for these scales, so participants were clear about what these meant. The wording was adjusted on several questions; for example, one item stated that ‘I am afraid of people finding out that I have a denture’. Panel members felt this wording was too strong and that ‘worry’ is a better description, so the item was adjusted to ‘I worry about people finding out that I have a denture’. Other questions were deemed too vague or unclear and were adjusted accordingly. Overall, however, panel members reported that the scale was easy to follow. Any recommended changes were directed to making questions more meaningful and enabling a wider range of responses. Positively worded items were also included to reflect the positive benefits of having a removable denture.

The final draft of the P‐DEQ has 5 sections (Table 1) comprised of 34 items covering Body Function, Impacts on daily activities, Emotional impacts, Participation Impacts and Participation restrictions. The P‐DEQ measures the positive and negative impacts of living with a removable denture; specifically, this includes impacts on daily body functions (chewing food, affected taste and speech), impacts on daily activities (impacts on speaking in public, eating out and looking after the denture), emotional impacts (embarrassment, feeling older, feeling self‐conscious) and impacts on participation (feeling comfortable in social situations, hiding the denture or being worried about being seen with the denture) to more extreme participation restrictions (e.g., avoiding social gatherings, talking to friends and intimacy). The P‐DEQ therefore maps how changes in the bodily functions associated with a removable denture may or may not, in extreme cases, lead to restrictions on social life.

**TABLE 1:** The P‐DEQ impact scale.

Section type	Descriptor	Number of items	Scale
Body Function	Difficulty chewing, food getting stuck, altered taste, affecting speech, discomfort	5	6‐point frequency response (1. All the time 2. Very often 3. Fairly often 4. Occasionally 5. Hardly ever 6. Never)
Impacts on daily activities	Speaking in public, eating in public, managing the denture out of home, participating in hobbies, avoiding foods, removing denture to eat	6	7‐point likert response (1. Strongly agree 2. Agree 3. Somewhat agree 4. Neutral 5. Somewhat disagree 6. Disagree 7. Strongly disagree)
Emotional impacts	Embarrassment, avoidance, self‐conscious, feeling more like myself, smiling, don't think about it, worry, feeling older, feeling younger, shame, happiness, confidence, improved appearance	13	As above
Participation			
Impacts on participation	Feeling comfortable in social situations, worry about others finding out, worried about what people would think, feeling less attractive, hiding the denture, worried about being seen with the denture, comfortable having photographs because of denture	7	As above
b Restrictions on Participation	Avoid social gatherings, avoiding talking to friends about their denture, avoiding intimacy	3	As above
		34	Total

The numbers of items for the P‐DEQ and response formats are given in Table 1. Wherever possible we maintained the language used by participants to describe the impact and experience of living with a removable denture. Response options for the Body Function—Impacts on daily activities sub‐scale are on a 6‐point frequency‐based Likert Response format (1 All the time, 2 Very often, 3 Fairly often, 4 Occasionally, 5 Hardly ever, 6 Never). All other sub‐scales follow a 7‐point Likert Scale (1 Strongly agree, 2 Agree, 3 Somewhat agree, 4 Neutral, 5 Somewhat disagree, 6 Disagree, 7 Strongly disagree). These scales were advised by participants in the focus groups, after review from team members which included social scientists and dental healthcare professionals. Some items are worded positively to reflect the positive benefit that participants reported from having a denture compared to having a gap in their teeth.

Personal and environmental factors (see Figure 3) involved asking specific questions about the location and type of denture, reasons for having a denture, the main reason for having a denture and dental attendance (Table 2). We also designed a scale on the advice of the dentists in the team (NM and GMc) focusing on the personal relationship between the individual and their denture on several properties of the denture; this scale we termed MYDenture. It was felt at the time that this subscale could become especially useful for practitioners and patients in all appointments (Table 2, see Online Appendix S1 for full questionnaire). We report on the performance of this subscale alongside the subscales of the P‐DEQ in what follows.

**TABLE 2:** Additional items on the questionnaire.

Section type	Descriptor	Number of items	Scale
**Personal Factors**			
Location of denture	A complete upper, lower/A partial upper denture or removable bridge or partial lower denture or removable bridge.	1	N/A
Your denture	Is this your first denture or not, how many dentures before this one, How long have you been wearing dentures, How long have you been wearing your current denture for, What is the main reason for having a denture.		N/A
Reasons for having a denture	Tooth loss (decay, gum disease, accident, genetic)		N/A
Main reason for having a denture	Eat better, smile better, avoid embarrassment, speak better, be more attractive, resemble natural teeth, resolve problems		Check all that apply
Dental attendance	How often do they visit the dental professional?	1	At least every 3 months At least every 6 months At least once every year At least once every 2 years Less frequently than every 2 years Only when having trouble Never
MYDenture	Comfort, fit, security, replacement, being part of them, natural, disappointment		Check all that apply
**Environmental Factors**			
Demographics	Gender, gender at birth, age, ethnicity, occupation	5	N/A
**Global Scales**			
Global oral health	Overall, how would you rate your oral health	1	1. Excellent, 2. Very Good, 3. Good, 4. Fair, 5. Poor, 6. Very Poor.
Global health	Overall, how would you rate your general health	1	1. Excellent, 2. Very Good, 3. Good, 4. Fair, 5. Poor, 6. Very Poor.
Happiness with denture	Overall, I am happy with my denture.	1	1. Strongly agree 2. Agree 3. Somewhat agree 4. Neutral 5. Somewhat disagree 6. Disagree 7. Strongly disagree
Effect of denture on quality of life	Overall, how does your denture affect your quality of life	1	1. Very Positive, 2. Fairly positive, 3. Fairly negative, 4. Very negative.

### 3.1 Phase 2 Cross‐Sectional Validation

There was high initial engagement from Ipsos PanelOne, with an initial 80%–90% response rate from invited panellists. However, given strict quality checks *to ensure all participants had a removal partial denture* (in the form of uploading a photograph of their denture). A significant number of participants were subsequently disqualified. Consequently, the final response rate for the UK was 166 out of 400 (41.5%) participants in the US and 144 out of 800 (18.0%). The remaining 58 from the UK and 80 from the US were secured through external panels. The data for the UK and the US samples are provided in Tables 3, 4, 5, 6.

**TABLE 3:** Demographic characteristics of UK sample.

	% (*n*)
Gender	
Female	48.7 (109)
Male	51.3 (115)
Age group	
18–34	14.3 (32)
35–54	38.3 (88)
55–74	37.0 (83)
75+	26.8 (60)
Ethnic group	
English/Welsh/Scottish/Northern Irish/British	89.3 (200)
Irish	2.2 (5)
White other	0.4 (1)
Black and White Caribbean	0.9 (2)
Black and White African	1.8 (4)
Asian	3.6 (9)
Arab	0.9 (2)
Occupational status	
Employed full‐time	54.0 (121)
Employed part‐time	5.8 (13)
Self employed	4.5 (10)
Unemployed but looking for a job	1.8 (4)
Unemployed long‐term sick/disabled	4.5 (10)
Full‐time parent, homemaker	4.5 (10)
Retired	25.0 (56)

**TABLE 4:** UK sample occupational classification.

Social class	% (*n*)
High	6.3 (14)
Middle	17.4 (39)
Low	76.3 (171)

Table 7 provides a summary of the descriptive statistics for the total scores, for the P‐DEQ and the My Denture sub‐scales. The Total P‐DEQ score comprises of 34 items with a potential range of 34–238. As can be seen from Table 7, US participants had a slightly higher mean score (156.1, SD 30.4) compared to the UK participants (153.4, SD 27.6). The Emotional Function score is made up of 13 items with a potential range 13–91 with the US group reporting a slightly higher mean score (63.4, SD 13.3) than the UK group (61.5, SD 14.1). Overall, the mean scores are similar with minor variations.

The body function sub‐scale was measured on a slightly different scale to the other sub‐scales. This scale (Table 8) is measured on a 6‐point frequency scale, where a lower mean score indicates the problem is experienced more frequently (1 = All the time, 2 Very often, 3 Fairly often, 4 Occasionally, 5 Hardly ever, 6 Never). The mean score for the body function sub‐scale for both the UK and US samples was 20.9 (SD 5.9) and 20.5 (SD 5.4) respectively indicating that participants were experiencing impacts on a ‘fairly often’ to ‘occasional’ frequency (Table 7). The highest frequency impact score was for ‘My denture affects my speech’ (Item 23), with the UK group reporting this problem to be less frequent (4.57, SD 1.33) compared to the US group (4.45, SD 1.34). The problem reported to be the most frequent was ‘Food gets stuck under my denture’ (Item 21) for both groups, with the US group (3.68, SD 1.35) reporting this at a slightly higher frequency than the UK group (3.52, SD 1.46). For item‐total correlation coefficients anything above 0.4 is considered fair and above 0.8 considered good. Item total correlations ranged from 0.58 (‘My denture causes me discomfort’) to 0.42 (‘My denture affects my sense of taste for some foods or drinks’) for the UK data and from 0.68 (‘My denture causes me discomfort’) to 0.45 (‘Food gets stuck under my denture’) for the US data.

In the current draft the P‐DEQ has 13 items related to emotional impacts (Table 7) rated on a scale of 1–7 (1. Strongly agree to 7. Strongly disagree). The lower the score the higher the impacts with a possible range from 13 to 91. The UK mean score was 61.5 (SD 14.5); the US mean was 63.4 (SD 13.3), indicating that the overall mean scores were neutral to disagree for emotional impact. Such means potentially mask significant ranges in scores of 77 (UK data) and 69 (US data). The impacts on daily abilities subscale were scored across 9 items on the same scale as that for emotional impacts. Scores could range from 9 to 63, with a mean score for the UK data of 30.4 (SD 7.2) and 29.6 (SD 6.5) for the US data. These mean scores indicate that the scores for both sets of data were between somewhat agree and neutral for impacts. Once again, however, the scores had a range of 33 (UK data) to 31 (US data). Impacts on social participation were measured along the same response format (Table 8) with 7 items examining impacts on daily participation and 3 looking at severe restrictions on daily life.

For impacts on participation the mean scores for the UK and the US data were quite close 32.5 (SD 8.9) and 32.8 (SD 8.2) respectively (Table 7). These means indicate neutral to somewhat disagree in both samples that the denture is having an impact on daily participation. There were once more significant ranges in scores of between 42 (UK data) and 39 (US data). In relation to restrictions on participation in daily life there are 3 items with a score that can range from 3 to 21. The lower the score the higher the restrictions in social participation. The mean for the UK was 14.8 (SD 4.9) and for the US it was 15.3 (SD 4.6) demonstrating mild restrictions on social participation with the average score indicating neutral to somewhat disagreement with impacts on social participation. Within the samples there are significant variations, some participations experiencing severe restrictions on daily life and others experiencing none (UK and US ranges both 18).

The internal reliability (consistency) of the scale was assessed by examining item‐total correlations, sub‐scale total correlations and Cronbach's alpha coefficients. As with the body function sub‐scale for item‐total correlation coefficients anything above 0.4 is considered fair and above 0.8 considered good. Almost all remaining items for both the UK and the US data had correlations above 0.4 to the total P‐DEQ score, with the exception of ‘I feel younger because of my denture’ (for both the UK and the US) and ‘I do not think about my denture when I am wearing it’, ‘I am worried about being seen without my denture’, ‘I am better able to speak in public because of my denture’, ‘I am able to eat in public with my denture’ for the US (Table 5).

**TABLE 5:** Demographic characteristics of the US sample.

	% (*n*)
Gender	
F	50.0 (114)
M	49.1 (112)
Non‐binary	0.9 (2)
Age	
18–34	24.2 (55)
35–54	43.0 (98)
55–74	29.9 (68)
75+	3.1 (7)
Ethnic group	
White or Caucasian (not Hispanic or Latino)	76.3 (173)
Black or African‐American (not Hispanic or Latino)	10.5 (24)
Asian/Pacific Islander	3.1 (7)
Native American, Alaska Native, Aleutian	3.1 (7)
Hispanic or Latino (White or Caucasian)	4.8 (11)
Hispanic or Latino (Black or African‐American)	0.4 (1)
Hispanic or Latino (all other races/multiple races)	0.9 (2)
Other	0.9 (2)
Employment status	
Employed full‐time	57.9 (132)
Employed part‐time	2.2 (5)
Self employed	4.8 (11)
Unemployed but looking for a job	3.5 (8)
Unemployed Long‐term sick/disabled	5.3 (12)
Full‐time parent, homemaker	6.6 (15)
Retired	18.0 (41)
Student/Pupil	1.3 (3)

**TABLE 6:** Occupational classification for the US data.

Occupational grouping	% (*n*)
Management, Business, Science and Arts	46.5 (106)
Service Occupations	9.2 (21)
Sales and Office	14.5 (33)
Natural Resources, Construction and Maintenance	3.1 (7)
Production, Transportation and Material Moving	1.3 (3)
Military	2.6 (6)
Prefer not to say	22.8 (52)

**TABLE 7:** Total score, extent and sub‐scale scores for the P‐DEQ and My Denture, in the UK and the US.

	Number of items	Potential range	Actual range	UK range	US range	UK mean	UK SD	US mean	US SD
**The P‐DEQ and subdomains**									
Total P‐DEQ	34	34–238	204.0	146.0	139.0	153.4	27.6	156.1	30.4
**P‐DEQ sub‐scales**									
Body function	5	5–35	30.0	25.0	25.0	20.9	5.9	20.5	5.4
Emotional impacts	13	13–91	77.0	77.0	69.0	61.5	14.1	63.4	13.3
Impacts on daily activities	9	9–63	54.0	33.0	31.0	30.4	7.2	29.6	6.5
**Participation**									
Impacts	7	7–49	42.0	42.0	39.0	32.5	8.9	32.8	8.2
b Restrictions	3	3–21	18.0	18.0	18.0	14.8	4.9	15.3	4.6
**Personal factors sub‐scale**									
MY Denture	7	7–49	42.0	34.0	35.0	18.9	6.1	17.0	8.2

**TABLE 8:** Body function sub‐scale item impacts for UK and US.

Item*	Body Function sub scale	UK data				US data			
		Mean	SD	Item impact	Item‐total correlation	Mean	SD	Item impact	Item‐total correlation
20	My denture has made it difficult to chew	4.16	1.54	215	0.56	4.05	1.44	222	0.67
21	Food gets stuck under my denture	3.52	1.46	261	0.44	3.68	1.35	268	0.45
22	My denture affects my sense of taste for some foods or drinks	4.40	1.51	190	0.42	4.19	1.48	204	0.59
23	My denture affects my speech	4.57	1.33	184	0.46	4.45	1.34	183	0.61
24	My denture causes me discomfort	4.27	1.43	221	0.58	4.15	1.31	239	0.68

*Note:* Based on scores 6 point frequency scale where 1 All the time, 2 Very often, 3 Fairly often, 4 Occasionally, 5 Hardly ever, 6 Never. * all significant at the 0.001 level.

**TABLE 9:** Mean scores, standard deviation and item impact scores four of the sub‐scales for UK and US data.

Item^a^		UK data				US data			
		Mean	SD	Item impact	Item‐total correlation	Mean	SD	Item impact	Item‐total correlation
**Emotional impacts**									
25	Sometimes I feel embarrassed because of my denture	4.35	1.95	149	0.79	4.43	1.91	147	0.81
26	I avoid talking about my denture to others	3.66	1.88	173	0.67	3.69	1.86	172	0.57
27	My denture makes me feel self‐conscious	4.24	1.98	170	0.60	4.28	1.89	167	0.72
28	I feel more like myself with my denture	5.34	1.57	052	0.56	5.60	1.41	042	0.51
29	I feel my denture helps me to smile	5.63	1.35	035	0.56	5.90	1.28	026	0.45
30	I do not think about my denture when I am wearing it	4.75	1.91	138	0.51	4.81	1.68	103	0.30
31	I worry about breaking my denture	3.50	1.94	203	0.45	3.59	1.77	201	0.42
32	I feel older because of my denture	4.41	1.82	138	0.76	4.46	1.83	147	0.77
33	I feel younger because of my denture	4.27	1.82	122	0.25	4.79	1.68	086	0.24
34	I feel ashamed because I have my denture	4.77	1.98	121	0.78	4.86	1.87	121	0.81
35	I feel happier because I have my denture	5.45	1.48	044	0.51	5.62	1.28	034	0.46
36	My denture gives me more confidence	5.52	1.46	049	0.51	5.71	1.29	032	0.49
37	I feel my denture has improved my appearance	5.60	1.35	030	0.48	5.78	1.27	028	0.45
**Impacts on daily activities**									
38	I feel more comfortable in social situations because I have a denture	5.56	1.34	035	0.49	5.72	1.25	027	0.40
39	I worry about people finding out I have a denture	4.48	2.03	140	0.77	4.52	1.91	150	0.69
40	I am worried about what people will think about me if they find out I have a denture	4.57	1.96	140	0.75	4.46	1.96	148	0.69
41	I feel less attractive because I have dentures	4.61	1.88	129	0.80	4.67	1.81	123	0.77
42	I feel I have to hide my dentures from other people	4.43	2.01	139	0.77	4.46	1.93	141	0.72
43	I am worried about being seen without my denture	3.30	1.94	187	0.41	3.32	1.89	191	0.28
44	Because of my denture I am more comfortable having my photograph taken	5.56	1.42	039	0.52	5.68	1.44	035	0.41
45	I am better able to speak in public because of my denture	5.34	1.51	052	0.41	5.51	1.38	026	0.32
**Participation (a) Impacts**									
46	I am able to eat in public with my denture	5.75	1.50	051	0.52	5.63	1.51	057	0.39
47	I find it difficult to look after my denture when I am away from home	4.48	1.83	112	0.62	4.63	1.71	120	0.60
48	I cannot take part in my hobbies because of my denture	5.51	1.66	059	0.49	5.45	1.68	075	0.61
49	I have to avoid certain foods because of my denture	3.89	2.09	201	0.65	3.54	1.91	205	0.64
50	I have to take my denture out in order to eat	5.09	1.94	118	0.55	4.89	1.99	142	0.65
**Participation (b) Restrictions**									
51	I do not attend social gatherings because I have a denture	5.48	1.72	080	0.67	5.55	1.66	073	0.68
52	I cannot talk to my friends about my denture	4.65	1.95	110	0.76	4.83	1.79	108	0.65
53	I avoid intimate situations because I have a denture	4.70	1.92	123	0.74	5.55	1.66	121	0.71

^a^ 7 point Likert response: 1. Strongly agree, 2. Agree, 3. Somewhat agree, 4. Neutral, 5. Somewhat disagree, 6. Disagree, 7. Strongly disagree. The lower the score, the higher the impacts on this scale.

**TABLE 10:** DEQ Sub‐scale with item total correlations UK and US data.

Sub‐scale	UK data	US data
	Pearson correlation**	Pearson correlation**
Body Function	0.60	0.76
Emotional impacts	0.92	0.87
Impacts on daily activities	0.84	0.88
Participation		
Impacts	0.92	0.86
b Restrictions	0.84	0.78

**All significant at the 0.001 level.

**TABLE 11:** DEQ Cronbach's Alpha (UK and US Data).

	Number of items	UK Data	US Data
		Cronbach's Alpha (*n*–224)	Cronbach's Alpha (*n*–224)
Total score	34	0.81	0.76
Sub‐scales			
Body function	5	0.88	0.85
Emotional Impacts	13	0.86	0.87
Impacts on daily activities	6	0.76	0.71
Participation			
Impacts	7	0.83	0.79
b Restrictions	3	0.83	0.84

**TABLE 12:** MY Denture Sub‐scale correlation with DEQ Total score and sub‐scales.

	UK Data	US Data
Sub‐scale	Correlation	Correlation
Body Function	−0.31**	−0.48**
Emotional impacts	−0.61**	−0.72**
Impacts on daily activities	−0.35**	−0.51**
Participation		
Impacts	−0.35**	−0.34**
b Restrictions	−0.27**	−0.21**
Total score	Correlation	Correlation
DEQ	−0.50**	−0.55**

**All significant at the 0.001 level.

**TABLE 13:** DEQ Total score correlations with global health scores.

Sub‐scale	UK data (*n*–224)	US data (*n*–224)
	Correlation	Correlation
Overall how would you rate your oral health?	−0.31**	−0.17**
Overall how would you rate your general health?	−0.23**	−0.08
Overall I am happy with my denture.	−0.66**	−0.52**
Overall how does your denture affect your quality of life?	−0.39**	−0.15*
To what extent, if any does your denture have an impact on your quality of life?	−0.61**	−0.39**

*Significant at the 0.05 level, **significant at the 0.001 level.

For item impacts across the sub‐scales the P‐DEQ demonstrated an interesting range of scores (Table 9). For the emotional impacts sub‐scale UK Data, the lowest item impact score was for ‘I feel my denture has improved my appearance’ and the highest was for ‘I worry about breaking my denture’. For the US data, the lowest score was for ‘I feel my denture helps me to smile’ and the highest score was for ‘I worry about breaking my denture’. For the impacts on daily activities sub‐scale, the UK item impact scores the lowest score was for ‘I feel more comfortable in social situations because I have a denture’ and the highest was ‘I am worried about being seen without my denture’. For the US data, the lowest score was for ‘I am better able to speak in public because of my denture’ and the highest was for ‘I am worried about being seen without my denture’. For the daily abilities sub‐scale, the item ‘I am able to eat in public with my denture’ had the lowest score and ‘I have to avoid certain foods because of my denture’ had the highest score in both samples. Finally, for the participation (b) restrictions sub‐scale the item impact scores for both samples indicated that the item ‘I do not attend social gatherings because I have a denture’ had the lowest score and the item ‘I avoid intimate situations because I have a denture’ had the highest score. Items that are negatively framed (describing a problem, worry or negative feeling) tend to have higher Item Impact scores. Conversely, positively framed items (describing a benefit or positive feeling) generally show lower Item Impact scores. This suggests ‘Item Impact’ likely reflects how much an item contributes to a scale measuring the negative consequences or difficulties associated with dentures. The spread of Item impact scores was broadly similar for UK and US participants, with the emotional impact and daily abilities sub‐scales showing the widest ranges with maximum impact scores reaching just over 200. For this analysis the MyDenture sub‐scale is treated as a ‘Personal Factor’ (Figure 3) and so is not included in the overall P‐DEQ score.

#### 3.1.1 Sub‐Scale Analysis

Summary scores were generated for each of the sub‐scales. The total score is the sum of all scores for the scale for each participant. Scores can range from 34 to 238 for the total score (Table 7). All correlations between the sub‐scales and the total scores were consistent and significant at the 0.001 level (Table 10). The highest correlations were between the P‐DEQ total scores, the emotional impacts sub‐scale (0.92) and the participation impacts sub scale (0.92). The highest correlation for the US sample was between the total P‐DEQ score and impacts on daily activities. Cronbach's alpha data (Table 11) demonstrate that in both the UK and US samples the P‐DEQ and sub‐scales have good to high levels of internal consistency. The Cronbach's alpha for the total item impacts score was good in the UK (0.81) and the US (0.76) with the Cronbach's alpha for the item sub scales ranging from 0.88 (Body Function sub scale) to 0.76 (Impacts on Daily Activities sub scale) in the UK and 0.87 (Emotional Impacts sub scale) to 0.71 (Impacts on Daily Activities sub scale) in the US data.

The My Denture sub‐scale demonstrated highly significant but moderate correlations with the total P‐DEQ score and its sub scales (Table 12). Correlations with the total P‐DEQ score were −0.5 for the UK and −0.55 for the US. For the item sub scales the MyDenture sub‐scales ranged from −0.61 (Emotional Impacts sub scale) to −0.27 (Participation Restrictions sub scale) in the UK data. For the US data item sub scale correlations ranged from −0.72 (Emotional Impacts sub scale) to −0.21 (Participation Restrictions sub scale). These correlations indicate that the more highly participants rated their denture in terms of how comfortable it is, how it fits, if it is secure, if it replaces their teeth well, feels part of them and looks natural, the lower the overall impacts associated with quality of life.

The total P‐DEQ score was correlated with a range of global health scores (Table 13). It is important to note that higher scores on the P‐DEQ indicate a more negative denture experience; this explains the negative correlations with positive statements about health and oral health in general. Correlation scores range from negligible to moderate and strong. In the UK data, correlations ranged from −0.23 (‘Overall how would you rate your general health?’) to −0.66 (‘Overall I am happy with my denture’) and were highly significant. In contrast, for the US data, the correlations range from −0.08 (‘Overall how would you rate your general health?’) to −0.52 (‘Overall I am happy with my denture’), with the general health item not being significantly related to P‐DEQ scores.

## 4 Discussion

The aim of this study was to report on the development and preliminary validation of the Partial Denture Experience Questionnaire (P‐DEQ), designed to measure the impacts of living with removable partial dentures. The P‐DEQ is the first measure to be developed from a comprehensive multi‐staged, multi‐method study of what it means to have teeth removed and replaced by a removable denture. It has been developed to relate to the ICF [31] to reflect changes in debates about disability and rehabilitation in general [32, 33, 34, 35, 36, 37]. We also adopted this model because the detailed qualitative analysis revealed that there were significant positive aspects to living with a denture [62] and that the core problems associated with living with tooth loss and dentures are closely related to the emotional impacts of tooth loss [49, 56, 57], the benefits of removable partial dentures and social participation. The findings are in keeping with previous research on total tooth loss and recovery with dentures [24, 49, 56, 57] but go beyond this to closely follow more recent debates on measuring oral health [32].

### 4.1 The Conceptual Development of the P‐DEQ

Measuring the benefits and difficulties associated with living with an oral device like a partial denture is challenging. All measures of OHRQoL are in some way functionalist in their orientation because they are based on similar underlying conceptualisations of the relationship between oral health conditions and their impacts on quality of life. Locker's model of oral health was explicitly based on an adaptation of the ICIDH [7, 21] which is itself an earlier version of the ICF [63]. The models are conceptually similar; they imply that disease or health conditions may or may not lead to handicap or decreased social participation. The principal difference between these models is that the ICF model is much more interactive, placing emphasis on individual and environmental factors that might intervene to either promote or reduce such negative outcomes. Both the OHIP‐EDENT, the OHIP and the P‐DEQ are similar because they are based on a functionalist idea of the body and so both include an evaluation of functional impacts (See Online Appendix S1, Tables S1 and S2). They differ in crucial respects, however. The P‐DEQ has a number of additional items under body functions and differs with its additional focus on emotional impacts (13 items as opposed to 2 under psychological function for the OHIP‐EDENT). In addition to this, the items are focused on the *interaction* between the participant and their environment rather than the individual experience of symptoms. In this key respect, the measures are quite different. For example, the OHIP‐EDENT asks ‘Have you been worried by dental problems?’ The P‐DEQ asks if participants ‘worry about people finding out that I have a denture’, or if they are ‘worried about what people will think about me if they find out I have a denture’. These impacts are therefore more focused on social participation than the OHIP‐EDENT, reflecting differences in the underlying conceptual model.

Qualitative research has shown that whilst the impacts of tooth loss can be very emotional [49], many patients value the benefits of their denture despite the challenges they might involve [62]. To reflect these findings, it is necessary to attempt to integrate both positive and negative experiences into the measurement impacts on daily life, reflecting earlier work on positive health [39] and the concept of positive wellbeing [24]. These ideas are usually tapped into through positively worded items in questionnaires such as these [38]. As we have found, however, incorporating these ideas is not straightforward.

Within the ICF framework, ‘Personal Factors’ are the intrinsic attributes of an individual that can influence their experience of health and disability [31]. They are contextual elements related to the individual themselves. While the ICF does not classify these factors in detail, it acknowledges their crucial role in shaping how a person experiences their functioning, activities and participation. In this study we developed a separate sub‐scale, proposed by dental professionals working on the team, seeking to measure the relationship between participants and their denture. It is suggested that the ‘My Denture’ sub‐scale could act as an important personal factor moving forward in the analysis of changes in living with removable partial dentures. Items within the ‘My Denture’ sub‐scale assess qualities such as comfort, fit, security and how natural the denture looks and feels. These are not objective clinical measures but are instead deeply personal evaluations. A participant who perceives their denture as ‘part of them’ (a highly personal appraisal) may very well experience fewer participation restrictions, even if they face some functional challenges like food getting stuck under their denture.

This is in keeping with qualitative research reporting that for some participants their denture has become part of who they are [62, 64]. A finding that hints at the possibility that some form of ‘homoeostatic’ balance between the individual, their environment and their denture might be an ideal point of measurement. In contrast someone who appraises their denture as ill‐fitting or unnatural may experience greater emotional impacts and social avoidance, regardless the clinical properties of the denture. The data from this study supports this conceptualisation in two ways. Firstly, the ‘My Denture’ sub‐scale demonstrated a significant negative correlation with the total P‐DEQ score and its sub‐scales, particularly the Emotional impacts sub‐scale (correlations of −0.61 in the UK and −0.72 in the US). This indicates that a more positive personal appraisal of the denture is strongly associated with fewer negative impacts. This personal evaluation may act as a mediating factor between the health condition (tooth loss and provision with a denture) and the individual's lived experience of disability and well‐being. Secondly, the scales for many of the items on the P‐DEQ are based on a response set sitting on a balanced seven‐point Likert scale (Table 9). The mid‐point of this scale is ‘4. neutral’, which could be interpretated as indicating a state of homeostasis.

The study findings also map onto recent debates about the importance of measuring positive health and social participation [32, 39]. The mean sub scores for the body function sub‐scale show that the experience of impacts in both samples was relatively mild, with food getting stuck under the denture being the more frequent impact (between Fairly often to Occasionally). Participants in both samples were experiencing low frequency impacts but nonetheless reported higher item impacts associated with worrying about their denture breaking and keeping it hidden from others in both samples. This provides empirical support for the idea of ‘prosthetic privacy’ [65] and potentially supports the recently discussed problem of ‘managing disclosure’ [62]. Further evidence of this might be related to the ambivalence with which participants from both samples reacted to positively worded items in the questionnaire. For example, mean scores indicated that participants strongly disagreed that they were happier that they had a denture, that it improved their appearance, and that it gave them more confidence. This provides important quantitative support for the idea that the context of living with a removable prosthesis is shaped by the experience of tooth loss [65].

### 4.2 Psychometric Properties of the P‐DEQ

The P‐DEQ is comprised of 34 items across 5 sub‐scales closely mapped onto the ICF [31]. The wide range of scores in each sample, coupled with their consistency across samples, indicates good content validity. Although the data provide some preliminary support for the idea that the P‐DEQ may be able to discriminate between different levels of impact, the focus of research moving forward will be to adjust the P‐DEQ to measure change. The range of responses to individual items suggests that the questionnaire is picking up significant variation in impact and so may be useful for this purpose. Further longitudinal testing will be required to examine this more carefully. This will also enable the elimination of unresponsive items and so help in shortening the questionnaire.

The item impact data and item‐total correlations demonstrate that emotional impacts and participation (a) impacts and (b) restrictions are highly correlated with the overall P‐DEQ score. Emotional responses most associated with the total scale scores are feeling embarrassed, ashamed and old. The findings also show that having to avoid certain foods, managing the denture away from home, and having to remove the denture when eating are strongly associated with total scores. The mean scores for these items indicate that food getting stuck under the denture is the most frequent daily problem. This indicates there is some across scale consistency; it also indicates some of the most common daily problems patients are confronted with. While these strong associations for emotional function and social participation echo the significant psychosocial impacts documented in the literature for complete denture wearers [49, 66], the P‐DEQ's validation with partial denture users provides a more nuanced understanding of this experience. This involves not only coping with tooth loss but also managing a prosthesis alongside remaining natural teeth.

The sub‐scale to item‐total correlations were all in the good‐very good range with the emotional impacts, impacts on daily activities and participation sub‐scales showing the strongest associations. These findings triangulate with existing qualitative and quantitative research into the emotional impact of tooth loss and what it means to live with a complete denture [49, 50, 56]. For partial denture wearers, the high correlation of these sub‐scales is particularly telling. It reflects a daily reality where functional difficulties, such as avoiding certain foods, are not merely practical inconveniences but are intrinsically linked to emotional distress, such as feeling embarrassed or ashamed, which can in extreme cases lead to social withdrawal (participation restrictions). The P‐DEQ seeks to capture how the constant management of the prosthesis, distinct from the experience of being fully edentulous, shapes the user's social and emotional world.

The underlying complexity of the experience makes measuring quality of life in these patients especially challenging. Not only are patients adapting to tooth loss, but they are also adapting to a removable prothesis along with the consequences this brings. At the same time, they may also be experiencing further deterioration in their gums and remaining dentition. Generic measures alone may not be able to capture many of these complex changes. Consequently, they will not be able to measure the benefits of different methods for managing these devices.

The results of this study provide strong preliminary evidence for the reliability of the P‐DEQ's sub‐scales, with Cronbach's alpha values meeting or exceeding the thresholds for acceptable reliability in both the UK and US samples. However, as psychometric theorists like Davenport et al. [67] caution, coefficient alpha is sensitive to test length in addition to the interrelatedness of items. Given that this initial version of the P‐DEQ contains a set of 34 items, the high reliability estimates may be influenced by the substantial number of items within the sub‐scales. This is an expected and acceptable outcome for this initial stage of validation. The primary goal of this cross‐sectional study is to establish the content validity and initial reliability of the full item set, developed directly from patient experiences. While this has been achieved, this study design does not provide the necessary evidence for item reduction. The process of eliminating items requires longitudinal data to assess how individual items perform over time, their sensitivity to change (responsiveness), to ensure that a shorter instrument maintains its robust psychometric properties.

Nonetheless these data remain preliminary. Further data on meaningfully important changes as well as item ratings from both clinicians and patients are also required. Future analysis might more effectively model the complex interplay between the prosthesis, the person and their environment. This could provide crucial insights into why individuals with clinically similar prostheses have vastly different outcomes and could help tailor interventions that address not just the function of the denture, but the patient's personal relationship with it. Although in this study we follow Classical Test Theory (CTT), research is also needed that will allow for a more detailed item‐level analysis, potentially using methods from Item Response Theory (IRT) [68] to examine item characteristics in detail. The objective of research moving forward will be to identify a core set of the most informative items, eliminating those that are redundant or show poor responsiveness to create a final, more concise version of the P‐DEQ. This process will seek to develop an optimised instrument that balances the high reliability established in this study whilst making the P‐DEQ as practically useful as possible for clinical practice. This will also be important regarding positive and negatively worded items. A key finding from the item‐level analysis is that these items perform very differently. Across the sub‐scales, positively framed items such as ‘I feel younger because of my denture’ or ‘I feel my denture has improved my appearance’, consistently demonstrated lower item‐total correlations and lower item impact scores compared to their negatively framed counterparts. These items, although intentionally included to capture the potential benefits of dentures, appear to be less reliable indicators of the overall denture experience measured by the P‐DEQ. This has previously been referred to as the ‘method effect’ or ‘wording effect’ [68]. Researchers have argued that the process of endorsing a positive statement can be different from endorsing a negative one, even when items are intended to measure the same underlying construct. Edelen et al. [68] found that positively worded (reverse‐scored) items loaded onto a separate factor from negatively worded items. Such items may represent a distinct, secondary dimension within the scale, which explains their weaker correlation with the other items. From the perspective of Classical Test Theory (CTT) [69], this lower inter‐item correlation will naturally result in lower item‐total correlations and could suppress the sub‐scale's overall Cronbach's alpha coefficient.

This method effect should not, however, be dismissed as mere statistical noise. There has been an ongoing debate about the issue of positive health in dental research [39]. It could be argued that these findings offer important quantitative support for the profound ambivalence that characterises the lived experience of wearing a denture, a point already raised in qualitative research [62, 69, 70]. Patients may simultaneously appreciate that the denture mitigates the negative consequences of tooth loss, while feeling no strong positive emotions like happiness or improved confidence about the prosthesis itself. The denture is often perceived as a necessary replacement to manage tooth loss, not as an enhancement that generates positive feelings. The P‐DEQ, by showing the different psychometric performance of positive and negative items, is attempting to capture the complex emotional states.

### 4.3 Limitations of the Study

While this study provides an essential first step in the development and validation of the P‐DEQ, several limitations should be considered when interpreting these findings. The initial qualitative work that informed the item generation for the P‐DEQ was conducted exclusively with participants in the UK. While the subsequent validation included a US sample, the foundational concepts may be UK‐centric. Previous work has shown that culture and place are important determinants of living with a removable denture, and so more cross‐cultural work may be required before it could be used more generally [64]. A significant limitation in the development process was that the panel testing to examine the face validity of the P‐DEQ was conducted exclusively with female participants. This lack of gender diversity at a crucial stage of refining the questions means the face validity from a male perspective was not assessed.

The quantitative validation involved data from two specific populations, recruited through online panels and involved participants from the UK and the US. These samples may not be fully representative of the broader population of removable partial denture wearers, particularly those who are not easy to recruit online, who might be from lower socioeconomic backgrounds, or in poorer general health. The population may also be relatively healthy and so the full spectrum of experience of living with tooth loss and a denture may not be fully represented. These data are cross‐sectional and whilst this is sufficient for assessing initial reliability it cannot provide the necessary evidence for item reduction: the data capture a single moment in time and cannot assess the P‐DEQ's ability to measure change or its responsiveness to interventions. The findings establish preliminary reliability and construct validity, but further testing is required before the P‐DEQ can be considered a fully validated instrument.

## Author Contributions

Barry J. Gibson contributed to all aspects of conceptualization, design, data collection, data acquisition, interpretation and analysis, drafted and critically revised the manuscript. Nicolas Martin contributed to the conceptualization, design, data acquisition, interpretation, drafting and analysis of both the qualitative study and the cross‐sectional validation. Bilal El‐Dhuwaib contributed to the conceptualization, data acquisition, interpretation and analysis of the qualitative study. Gerry McKenna contributed to the conceptualization, design, data acquisition, interpretation, drafting and analysis of both the qualitative study and the cross‐sectional validation. Sandra Clifford contributed to the conceptualization, design, data acquisition, interpretation, drafting and analysis of the cross‐sectional validation. Alastair Lomax contributed to the conceptualization, design, data acquisition, interpretation, drafting and analysis of the cross‐sectional validation. Sarah R. Baker contributed to the conceptualization, design, data acquisition, interpretation, drafting and analysis of both the qualitative study and the cross‐sectional validation. All authors gave final approval and agreed to be accountable for all aspects of the work.

## Funding

This work was supported by Haleon Ltd.

## Conflicts of Interest

The authors declare no conflicts of interest.

## Supporting information


**Data S1:** ger70063‐sup‐0001‐OnlineAppendix‐1.docx.

## Data Availability

The data that support the findings of this study are available from the corresponding author upon reasonable request.
