# Supplementary material for: The Development and Validation of the Partial Denture Experience Questionnaire (P‐DEQ): Reliability and Validity
Source: Gerodontology. 2026 Mar 5;43(3):539–57. doi: 10.1111/ger.70063 (PMC13498917; doi:10.1111/ger.70063)
Supplement: Supplementary file 1 [file GER-43-539-s001.docx]

Online Appendix 1

Figure 1 Locker’s Model of Oral Health

**Source: Locker**

**Table 1 The OHIP-EDENT***

| **Conceptual domain/Items** | **Response format** |
| --- | --- |
| **1. Functional limitation** | |
| Have you had difficulty chewing any foods because of problems with your teeth, mouth or dentures? | 0. never, 1. Hardly ever, 2. Occasionally, 3. Fairly Often, 4. Very Often. |
| Have you had food catching in your teeth or dentures? | 0. never, 1. Hardly ever, 2. Occasionally, 3. Fairly Often, 4. Very Often. |
| Have felt that your dentures have not been fitting properly? | 0. never, 1. Hardly ever, 2. Occasionally, 3. Fairly Often, 4. Very Often. |
| **2. Physical Pain** | |
| Have you found it uncomfortable to eat any foods because of problems with your teeth, mouth or dentures? | 0. never, 1. Hardly ever, 2. Occasionally, 3. Fairly Often, 4. Very Often. |
| Have you had sore spots in your mouth? | 0. never, 1. Hardly ever, 2. Occasionally, 3. Fairly Often, 4. Very Often. |
| Have you had uncomfortable dentures? | 0. never, 1. Hardly ever, 2. Occasionally, 3. Fairly Often, 4. Very Often. |
| **3. Psychological discomfort** | |
| Have you been worried by dental problems? | 0. never, 1. Hardly ever, 2. Occasionally, 3. Fairly Often, 4. Very Often. |
| Have you been self-conscious because of your teeth, mouth or dentures? | 0. never, 1. Hardly ever, 2. Occasionally, 3. Fairly Often, 4. Very Often. |
| **4. Physical Disability** | |
| Have you had to avoid eating some foods because of problems with your teeth, mouth or dentures? | 0. never, 1. Hardly ever, 2. Occasionally, 3. Fairly Often, 4. Very Often. |
| Have you been unable to eat with your dentures because of problems with them? | 0. never, 1. Hardly ever, 2. Occasionally, 3. Fairly Often, 4. Very Often. |
| Have you had to interrupt meals because of problems with your teeth, mouth or dentures? | 0. never, 1. Hardly ever, 2. Occasionally, 3. Fairly Often, 4. Very Often. |
| **5. Psychological Disability** | |
| Have you been upset because of problems with your teeth, mouth or dentures? | 0. never, 1. Hardly ever, 2. Occasionally, 3. Fairly Often, 4. Very Often. |
| Have you been a bit embarrassed because of problems with your teeth, mouth or dentures? | 0. never, 1. Hardly ever, 2. Occasionally, 3. Fairly Often, 4. Very Often. |
| **6. Social Disability** | |
| Have you avoided going out because of problems with your teeth, mouth or dentures? | 0. never, 1. Hardly ever, 2. Occasionally, 3. Fairly Often, 4. Very Often. |
| Have you been less tolerant of your partner or family because of problems with your teeth, mouth or dentures? | 0. never, 1. Hardly ever, 2. Occasionally, 3. Fairly Often, 4. Very Often. |
| Have you been irritable with other people because of problems with your teeth, mouth or dentures? | 0. never, 1. Hardly ever, 2. Occasionally, 3. Fairly Often, 4. Very Often. |
| **7. Handicap** | |
| Have you been unable to enjoy other people’s company as much because of | 0. never, 1. Hardly ever, 2. Occasionally, 3. Fairly Often, 4. Very Often. |
| Have you felt that life in general was less satisfying because of problems with your teeth, mouth or dentures? | 0. never, 1. Hardly ever, 2. Occasionally, 3. Fairly Often, 4. Very Often. |

*Developed to fit Locker’s Model of Oral Health response options are from 0 (never) to 4 (very often).

Table 2 The PQL**

| **Conceptual Domain/Items** | **Response format** |
| --- | --- |
| **Prosthetic fit** | |
| What do you think about the fit of your upper/lower prosthesis? | 1. Very good, 2. Good, 3. Acceptable, 4 Bad, 5. Very Bad |
| **Chewing** | |
| Do you have to be careful about what you eat or drink owing to the quality of your prosthesis? | 1. No Never, 2. Yes but very occasionally, 3. Yes, Sometimes, 4. Yes, nearly always when I drink or eat, 5. I can’t eat with the prothesis in my mouth. |
| **Foreign Body** | |
| Do you feel the prosthesis is some kind of foreign body in your mouth or does it seem to be integrated in it? | 1. Completely integrated, as though it were my own, 2. I have become adapted to it and do not notice it, 3. I don’t seem able to adapt even though I wear it all the time, 4. I can’t adapt to it and I hardly ever wear it, 5. I never wear it because I can stand the feeling. |
| Aesthetics | |
| Do you think that the prosthesis has changed the aspect of your smile? | 1. Yes, a lot, 2. Yes, slightly 3. It’s more or less the same, 4. I think it’s worse, 5. It’s much worse. |
| **Communication** | |
| Do you avoid speaking to people because of the prosthesis? | 1. Never, 2. Rarely, 3. Sometimes, 4. Often, 5. Always. |
| **Realism of prosthesis** | |
| Do you think people realise you are wearing a prosthesis? | 1. Never, 2. Rarely, 3. Sometimes, 4. Often, 5. Always. |
| **Unnoticeability** | |
| Do you try to hide the fact that you are wearing a prosthesis? | 1. Never, 2. Rarely, 3. Sometimes, 4. Often, 5. Always. |
| **Hygiene** | |
| Do you think taking hygienic care of your prosthesis is easy? | 1. Very easy, 2. Easy, 3. Neither easy nor difficult, 4. Difficult, 5. Very difficult. |
| **Food impaction** | |
| Do you feel food getting impacted as a result of your prosthesis? | 1. Never, 2. Rarely, 3. Sometimes, 4. Often, 5. Always. |
| **Functional Comfort** | |
| Do you feel at ease with your prosthesis with respect to the usual functions of your mouth: eating, speaking, smiling…? | 1. I feel completely at ease, 2. I am fairly at ease, 3. I don’t feel too bad, 4. I feel a bit uncomfortable, 5. I feel very uncomfortable. |
| **Self-confidence** | |
| Does your prosthesis make you feel self-confident in your daily life? | 1. Yes, I feel very confident, 2. Yes, I feel fairly confident, 3. Neither one nor the other, 4. I don’t always wear it because I don’t feel very confident wearing it, 5. I hardly ever use it because I never feel confident when I’m wearing it. |

**Table 3 Preliminary item development and first full draft of P-DEQ**

| Preliminary Questionnaire Items | P-DEQ items for Cross-Sectional Testing |
| --- | --- |
| The following questions are about your denture.  **Personal factors**  1. On a scale of 1-10, how comfortable are your dentures?  2. My denture is comfortable.  3. On a scale of 1-10 how would you rate the ‘fit’ of your denture?  4. My denture fits well.  5. On a scale of 1-10, how stable is your denture?  6. My denture is stable.  7. On a scale of 1 to 10 how well does the denture fill the gap in your mouth?  8. My denture fills the gap in my mouth well.  9. On a scale of one to 10 how intrusive is your denture?  10. My denture is intrusive.  11. On a scale of one to 10 closely would you say your denture appears like your normal/natural teeth?  12. My denture looks like my natural teeth.  **Impact measure**  **Body functions**  1. My denture gives me difficulty in chewing.  2. Food gets stuck under my denture.  3. My denture has diminished my sense of taste.  4. I have to speak around my denture.  5. My denture impacts on the sound of my speech.  6.My denture has enabled me to smile like I could before.  7. My denture causes me pain.  8. I feel embarrassed because of my denture.  9. I feel less self-conscious because of my denture.  10. I feel normal with my denture in.  11. I constantly worry about breaking my denture.  12. I feel old(er) because of my denture.  13. I feel less ashamed now because I have my denture.  14.I put myself down because of my denture.  15. I am happier now I have my denture  16. I am disappointed with my denture  **Impacts on daily activities**  17. I can fit better into social situations because of my denture.  18. I am afraid of people finding out that I have a denture.  19. I am worried about what people will think about me if they find out I have a denture.  20. I feel less attractive because I have dentures.  21.I have to hide my dentures from other people.  22. I am worried about being seen without my denture  **Denture and confidence (confidence is multidimensional - if we had several questions - this might link the body structures and functions with disability?**  23. My denture gives me more confidence in chewing.  24. My denture gives me more confidence in smiling.  25. My denture gives me more confidence when speaking(?).  26. I am more confident in social situations when I have my denture.  27. I am more confident in my appearance because of my denture.  28. I am more confident at having my photograph taken because of my denture.  **Participation (positive and negative]**  29. I feel better able to speak in public because of my denture.  30. I am able to eat in public groups with my denture.  31. I can look after my denture as a normal part of my daily routine.  32. I am no longer able to engage with my hobbies because of my denture.  33.I am very self conscious of having to manage my denture in places outside my home.  **Participation Restrictions**  34. I have to avoid certain foods when I am wearing my denture.  35. I have to take my denture out in order to eat.  36. I no longer attend the dentist now I have a denture.  37. I no longer attend large social gatherings because I have a denture.  38. I have withdrawn from certain social situations because I have a denture.  39. I cannot talk to my family and friends about my denture.  40. I avoid relationships because I have a denture.  41. I am unable to be intimate with my partner because I have a denture.  **Global Oral Health Questions**  1. My denture is part of me now.  2. I am able to forget that I have a denture.  **Global Health Rating.**  1. Overall how would you rate your oral health without your denture?  2. Overall how would you rate your oral health with your denture?  3. Overall I am happy with my denture.  4. Overall how do you rate your ability to interact with others?  5. Overall how do you rate your ability to participate in daily life?  6. Overall how do the problems with your denture affect your quality of daily life? | **Impact Measure P-DEQ**  **Body function**   1. My denture has made it difficult to chew. 2. Food gets stuck under my denture. 3. My denture affects my sense of taste for some foods or drinks. 4. My denture affects my speech. 5. My denture causes me discomfort.   **Emotional impacts**   6. Sometimes I feel embarrassed because of my denture. 7. I avoid talking about my denture to others. 8. My denture makes me feel self-conscious. 9. I feel more like myself with my denture. 10. I feel my denture helps me to smile. 11. I do not think about my denture when I am wearing it. 12. I worry about breaking my denture. 13. I feel older because of my denture. 14. I feel younger because of my denture. 15. I feel ashamed because I have my denture. 16. I feel happier because I have my denture. 17. My denture gives me more confidence. 18. I feel my denture has improved my appearance.   **Impacts on daily activities**   19. I feel more comfortable in social situations because I have a denture. 20. I worry about people finding out that I have a denture. 21. I am worried about what people will think about me if they find out I have a denture. 22. I feel less attractive because I have dentures. 23. I feel I have to hide my dentures from other people. 24. I am worried about being seen without my denture. 25. Because of my denture I am more comfortable having my photograph taken.   **Participation a) impacts**   26. I am better able to speak in public because of my denture. 27. I am able to eat in public with my denture. 28. I find it difficult to look after my denture when I am away from home. 29. I cannot take part in my hobbies because of my denture. 30. I have to avoid certain foods because of my denture 31. I have to take my denture out in order to eat.   **Participation b) Restrictions**   32. I do not attend social gatherings because I have a denture. 33. I cannot talk to my friends about my denture. 34. I avoid intimate situations because I have a denture.   **Global Health Ratings.**   1. Overall, how would you rate your oral health? 2. Overall, how would you rate your general health? 3. Overall, I am happy with my denture. 4. Overall, how does your denture affect your quality of life? 5. To what extent, if any, has does your denture have an impact on your quality of life?   **The MYDenture Subscale**   1. My denture is comfortable. 2. My denture fits well. 3. My denture feels secure. 4. My denture replaces my missing teeth well. 5. My denture is a part of me. 6. My denture looks natural. 7. I am disappointed with my denture. |

**Table 4 Summary of Participants involved in the narrative interviews^*^.**

| **Participant number and name** | **Age** | **Sex** | **Occupation** |
| --- | --- | --- | --- |
| Participant 1 - Keith | 46 | Male | Warehouse Manager |
| Participant 2 – Betty | 44 | Female | Disability Support Advisor |
| Participant 3 – Penny | 61 | Female | Nurse (retired) |
| Participant 4 - Bill | 52 | Male | Car Mechanic |
| Participant 5 - Emma | 46 | Female | Care Home Assistant |
| Participant 6 - Florence | 24 | Female | University Student |
| Participant 7 - Rose | 26 | Female | Nurse |
| Participant 8 - Lyana | 22 | Female | University Student |
| Participant 9 - Venus | 71 | Female | School Teacher (Retired) |
| Participant 10 - Shay | 64 | Female | Manager (retired) |
| Participant 11 - Ben | 75 | Male | Gardener (retired) |
| Participant 12 - Paul | 64 | Male | College Lecturer (retired) |
| Participant 13 - Alex | 86 | Male | Metal Caster (retired) |
| Participant 14 - Carrie | 71 | Female | Receptionist (retired) |
| Participant 15 - Michael | 71 | Male | Mental Health Nurse (retired) |
| Participant 16 - James | 73 | Male | Youth Worker (retired) |
| Participant 17 - Bobby | 66 | Male | Engineer |
| Participant 18 - Mark | 69 | Male | Engineer (retired) |
| Participant 19 - Stephen | 75 | Male | Teacher (retired) |
| Participant 20 - Roger | 71 | Male | Builder |

*** All Names are pseudonyms.**

**Full Questionnaire**

**Section 1 – Demographics**

The following questions are about your demographic details.

1. **Which of the following best describes your gender?**

☐	Man

☐	Woman

☐	Non-binary

☐	My gender is not listed

☐	I would prefer not to answer

2. **Not all people feel that the sex they were described as at birth matches how they feel about themselves today. So, to check, at birth, were you described as:**

☐	Female

☐	Male

☐	Prefer not to ~~say~~ answer

3. **What is your age?**

SW: HIDDEN QUESTION. AUTOCODE FROM AGE. SINGLE CODE.

☐	Under 18

☐	18-24

☐	25-34

☐	35-44

☐	45-54

☐	55-64

☐	65-74

☐ 75-80

☐ 81+

**4._UK FOR UK SHOW FOLLOWING:**

**What is your ethnic group?**

White

☐	English, Welsh, Scottish, Northern Irish or British

☐	Irish

☐	Gypsy or Irish Traveller

☐	Roma

☐	Any other White background, please write in below:

________________________

Mixed or Multiple ethnic groups

☐	White and Black Caribbean

☐	White and Black African

☐	White and Asian

☐	Any other Mixed or Multiple background, please write in below:

________________________

Asian or Asian British

☐	Indian

☐	Pakistani

☐	Bangladeshi

☐	Chinese

☐	Any other Asian background, please write in below:

________________________

Black, Black British, Caribbean or African

☐	Caribbean

☐	African background, please write in below

☐	Any other Black, Black British or Caribbean background, please write in below

________________________

Other ethnic group

☐	Arab

☐	Any other ethnic group, please write in below

________________________

☐	Prefer not to ~~say~~ answer

**4_US.** **FOR USA SHOW FOLLOWING:**

**Which of the following best describes you?**

☐	White or Caucasian (not Hispanic or Latino)

☐	Black or African-American (not Hispanic or Latino)

☐	Asian/Pacific Islander

☐	Native American, Alaska Native, Aleutian

☐	Hispanic or Latino (White or Caucasian)

☐	Hispanic or Latino (Black or African-American)

☐	Hispanic or Latino (all other races/multiple races)

☐	Other

☐	Prefer not to ~~say~~ answer

**5a. What is your current employment status?**

**[UK list]**

_1 Employed full-time

_2 Employed part-time

_3 Self employed

_4 Unemployed but looking for a job

_5 Unemployed and not looking for a job/Long-term sick or disabled

_6 Full-time parent, homemaker

_7 Retired

_8 Student/Pupil

_9 Prefer not to answer

**[USA list]**

_1 Employed full-time

_2 Employed part-time

_3 Self employed

_4 Unemployed but looking for a job

_5 Unemployed and not looking for a job/Long-term sick or disabled

_6 Full-time parent, homemaker

_7 Retired

_8 Student/Pupil

_9 Military
_10 Prefer not to answer

**5b. In which of the below categories does your occupation fall? If retired or unemployed, please indicate the category closest to your previous occupation.**

**[Insert UK list for UK, UK01OCCR]**

**Insert USA list for USA]**

**Include “Prefer not to ~~say~~ answer” option**

**5c. At the end of the survey, we will need to validate your type of denture. To do this, we will ask you to remove your denture and take a photo of it. You will have the option to upload it immediately or return to the link later at your convenience.**

☐	Yes, I agree to upload a photo of my denture at the end of the survey

☐	No, I do not agree to upload a photo of my denture at the end of the survey **[TERMINATE]**

**Main Questionnaire**

**Thank you for agreeing to fill in this questionnaire. The questions are designed to assess what it is like to live with a denture. Please read all the questions and tick the one box per question that applies most to you. There are no right or wrong answers, and all the answers you provide are confidential.**

**Section 2 – Details about your denture(s)**

The following questions (6-12) are about the location and characteristics of your denture(s).

6. **Which teeth does your denture(s) replace? (tick all that apply)**

☐ Top jaw - front of mouth

☐ Top jaw - back of mouth

☐ Bottom jaw - front of mouth

☐ Bottom jaw - back of mouth

7. **Is your current denture, your first denture?**

☐	Yes

☐	No

8. **How many dentures have you had before this one?**

**[ASK IF Q7=NO]**

☒	0

☐	1

☐	2

☐	3

☐	4

☐	5

9. **How long have you been wearing denture(s)?**

☐	Less than six months

☐	Six months to one year

☐	One year to three years

☐	Three years to five years

☐	Five years to ten years

☐	More than ten years

10. **How long have you had your current denture(s) for?**

☐	Less than six months

☐	Six months to one year

☐	One year to three years

☐	Three years to five years

☐	Five years to ten years

☐	More than ten years

11. **What is the main reason for having your denture(s)?**

☐ Tooth loss – tooth decay

☐ Tooth loss – gum disease

☐ Tooth loss – accident/trauma

☐ Teeth never came through (genetic)

☐ Other

**Please specify if answered ‘other’**

____________________________

12. **The main reason I have a denture to fill the gap(s) is to … (please tick your top 3 reasons)**

☐ Eat better

☐ Smile better

☐ Avoid social embarrassment

☐ To speak better

☐ To be more attractive

☐ To resemble my natural teeth

☐ To resolve long lasting dental problems

☐ Other

**Please specify if answered ‘other’**

___________________________

**Section 3 – Feelings towards your denture**

The following questions (13-19) reflect how you feel about your denture in general:

Thinking about your denture(s) in general to what extent would you agree or disagree with the following statements:

**[RANDOMISE STATEMENTS]**

13. **My denture is comfortable.**

☐ Strongly agree

☐ Agree

☐ Somewhat agree

☐ Neutral

☐ Somewhat disagree

☐ Disagree

☐ Strongly disagree

14. **My denture fits well.**

☐ Strongly agree

☐ Agree

☐ Somewhat agree

☐ Neutral

☐ Somewhat disagree

☐ Disagree

☐ Strongly disagree

15. **My denture feels secure.**

☐ Strongly agree

☐ Agree

☐ Somewhat agree

☐ Neutral

☐ Somewhat disagree

☐ Disagree

☐ Strongly disagree

16. **My denture replaces my missing teeth well.**

☐ Strongly agree

☐ Agree

☐ Somewhat agree

☐ Neutral

☐ Somewhat disagree

☐ Disagree

☐ Strongly disagree

17. **My denture is a part of me.**

☐ Strongly agree

☐ Agree

☐ Somewhat agree

☐ Neutral

☐ Somewhat disagree

☐ Disagree

☐ Strongly disagree

18. **My denture looks natural.**

☐ Strongly agree

☐ Agree

☐ Somewhat agree

☐ Neutral

☐ Somewhat disagree

☐ Disagree

☐ Strongly disagree

19. **I am disappointed with my denture.**

☐ Strongly agree

☐ Agree

☐ Somewhat agree

☐ Neutral

☐ Somewhat disagree

☐ Disagree

☐ Strongly disagree

**Section 4 – Changes in daily life**

The following questions (20-24) are about the way in which your denture(s) may have led to changes in your daily life.

Thinking about your denture(s) over the last month, how often have you experienced the following because of your denture:

**[RANDOMISE STATEMENTS]**

20. **My denture has made it difficult to chew.**

☐ All the time

☐ Very often

☐ Fairly often

☐ Occasionally

☐ Hardly ever

☐ Never

21. **Food gets stuck under my denture.**

☐ All the time

☐ Very often

☐ Fairly often

☐ Occasionally

☐ Hardly ever

☐ Never

22. **My denture affects my sense of taste for some foods or drinks.**

☐ All the time

☐ Very often

☐ Fairly often

☐ Occasionally

☐ Hardly ever

☐ Never

23. **My denture affects my speech.**

☐ All the time

☐ Very often

☐ Fairly often

☐ Occasionally

☐ Hardly ever

☐ Never

24. **My denture causes me discomfort.**

☐ All the time

☐ Very often

☐ Fairly often

☐ Occasionally

☐ Hardly ever

☐ Never

**Section 5 – How your denture makes you feel**

The following questions (25-37) are about the way your denture(s) makes you feel.

Thinking about your denture(s) over the last month to what extent would you agree or disagree with the following statements:

**[RANDOMISE STATEMENTS]**

25. **Sometimes I feel embarrassed because of my denture.**

☐ Strongly agree

☐ Agree

☐ Somewhat agree

☐ Neutral

☐ Somewhat disagree

☐ Disagree

☐ Strongly disagree

26. **I avoid talking about my denture to others.**

☐ Strongly agree

☐ Agree

☐ Somewhat agree

☐ Neutral

☐ Somewhat disagree

☐ Disagree

☐ Strongly disagree

27. **My denture makes me feel self-conscious.**

☐ Strongly agree

☐ Agree

☐ Somewhat agree

☐ Neutral

☐ Somewhat disagree

☐ Disagree

☐ Strongly disagree

28. **I feel more like myself with my denture.**

☐ Strongly agree

☐ Agree

☐ Somewhat agree

☐ Neutral

☐ Somewhat disagree

☐ Disagree

☐ Strongly disagree

29. **I feel my denture helps me to smile.**

☐ Strongly agree

☐ Agree

☐ Somewhat agree

☐ Neutral

☐ Somewhat disagree

☐ Disagree

☐ Strongly disagree

30. **I do not think about my denture when I am wearing it.**

☐ Strongly agree

☐ Agree

☐ Somewhat agree

☐ Neutral

☐ Somewhat disagree

☐ Disagree

☐ Strongly disagree

31. **I worry about breaking my denture.**

☐ Strongly agree

☐ Agree

☐ Somewhat agree

☐ Neutral

☐ Somewhat disagree

☐ Disagree

☐ Strongly disagree

32. **I feel older because of my denture.**

☐ Strongly agree

☐ Agree

☐ Somewhat agree

☐ Neutral

☐ Somewhat disagree

☐ Disagree

☐ Strongly disagree

33. **I feel younger because of my denture.**

☐ Strongly agree

☐ Agree

☐ Somewhat agree

☐ Neutral

☐ Somewhat disagree

☐ Disagree

☐ Strongly disagree

34. **I feel ashamed because I have my denture.**

☐ Strongly agree

☐ Agree

☐ Somewhat agree

☐ Neutral

☐ Somewhat disagree

☐ Disagree

☐ Strongly disagree

35. **I feel happier because I have my denture.**

☐ Strongly agree

☐ Agree

☐ Somewhat agree

☐ Neutral

☐ Somewhat disagree

☐ Disagree

☐ Strongly disagree

36. **My denture gives me more confidence.**

☐ Strongly agree

☐ Agree

☐ Somewhat agree

☐ Neutral

☐ Somewhat disagree

☐ Disagree

☐ Strongly disagree

37. **I feel my denture has improved my appearance.**

☐ Strongly agree

☐ Agree

☐ Somewhat agree

☐ Neutral

☐ Somewhat disagree

☐ Disagree

☐ Strongly disagree

**Section 6 – Your denture when you are with others**

The following questions (38-44) are about how you feel when you are with other people or in certain situations.

Thinking about your denture(s) over the last month, to what extent would you agree or disagree with the following statements:

**[RANDOMISE STATEMENTS]**

38. **I feel more comfortable in social situations because I have a denture.**

☐ Strongly agree

☐ Agree

☐ Somewhat agree

☐ Neutral

☐ Somewhat disagree

☐ Disagree

☐ Strongly disagree

39. **I worry about people finding out that I have a denture.**

☐ Strongly agree

☐ Agree

☐ Somewhat agree

☐ Neutral

☐ Somewhat disagree

☐ Disagree

☐ Strongly disagree

40. **I am worried about what people will think about me if they find out I have a denture.**

☐ Strongly agree

☐ Agree

☐ Somewhat agree

☐ Neutral

☐ Somewhat disagree

☐ Disagree

☐ Strongly disagree

41. **I feel less attractive because I have dentures.**

☐ Strongly agree

☐ Agree

☐ Somewhat agree

☐ Neutral

☐ Somewhat disagree

☐ Disagree

☐ Strongly disagree

42. **I feel I have to hide my dentures from other people.**

☐ Strongly agree

☐ Agree

☐ Somewhat agree

☐ Neutral

☐ Somewhat disagree

☐ Disagree

☐ Strongly disagree

43. **I am worried about being seen without my denture.**

☐ Strongly agree

☐ Agree

☐ Somewhat agree

☐ Neutral

☐ Somewhat disagree

☐ Disagree

☐ Strongly disagree

44. **Because of my denture I am more comfortable having my photograph taken.**

☐ Strongly agree

☐ Agree

☐ Somewhat agree

☐ Neutral

☐ Somewhat disagree

☐ Disagree

☐ Strongly disagree

**Section 7 – Daily activities**

The following questions (45-50) are about your denture(s) and your daily activities.

Thinking about your denture(s) over the last month, to what extent would you agree or disagree with the following statements:

**[RANDOMISE STATEMENTS]**

45. **I am better able to speak in public because of my denture.**

☐ Strongly agree

☐ Agree

☐ Somewhat agree

☐ Neutral

☐ Somewhat disagree

☐ Disagree

☐ Strongly disagree

46. **I am able to eat in public with my denture.**

☐ Strongly agree

☐ Agree

☐ Somewhat agree

☐ Neutral

☐ Somewhat disagree

☐ Disagree

☐ Strongly disagree

47. **I find it difficult to look after my denture when I am away from home.**

☐ Strongly agree

☐ Agree

☐ Somewhat agree

☐ Neutral

☐ Somewhat disagree

☐ Disagree

☐ Strongly disagree

48. **I cannot take part in my hobbies because of my denture.**

☐ Strongly agree

☐ Agree

☐ Somewhat agree

☐ Neutral

☐ Somewhat disagree

☐ Disagree

☐ Strongly disagree

49. **I have to avoid certain foods because of my denture**

☐ Strongly agree

☐ Agree

☐ Somewhat agree

☐ Neutral

☐ Somewhat disagree

☐ Disagree

☐ Strongly disagree

50. **I have to take my denture out in order to eat.**

☐ Strongly agree

☐ Agree

☐ Somewhat agree

☐ Neutral

☐ Somewhat disagree

☐ Disagree

☐ Strongly disagree

**Section 8 – Restrictions in participating**

The following questions (51-53) are about your denture(s) and participation in certain life situations.

Thinking about your denture(s) over the last month, to what extent would you agree or disagree with the following statements:

**[RANDOMISE STATEMENTS]**

51. **I do not attend social gatherings because I have a denture.**

☐ Strongly agree

☐ Agree

☐ Somewhat agree

☐ Neutral

☐ Somewhat disagree

☐ Disagree

☐ Strongly disagree

52. **I cannot talk to my friends about my denture.**

☐ Strongly agree

☐ Agree

☐ Somewhat agree

☐ Neutral

☐ Somewhat disagree

☐ Disagree

☐ Strongly disagree

53. **I avoid intimate situations because I have a denture.**

☐ Strongly agree

☐ Agree

☐ Somewhat agree

☐ Neutral

☐ Somewhat disagree

☐ Disagree

☐ Strongly disagree

**Section 9 – Summary health questions**

The following questions (54-58) are about your overall quality of life.

54. **Overall, how would you rate your oral health?**

☐ Excellent

☐ Very good

☐ Good

☐ Fair

☐ Poor

☐ Very poor

55. **Overall, how would you rate your general health?**

☐ Excellent

☐ Very good

☐ Good

☐ Fair

☐ Poor

☐ Very poor

56. **Overall, I am happy with my denture.**

☐ Strongly agree

☐ Agree

☐ Somewhat agree

☐ Neutral

☐ Somewhat disagree

☐ Disagree

☐ Strongly disagree

57. **Overall, how does your denture affect your quality of life?**

☐	Not at all

☐	A little

☐	Moderately

☐	Very much

☐	Extremely

58. **To what extent, if any, has does your denture have an impact on your quality of life? [SKIP IF NOT AT ALL AT 57]**

☐ Very positive

☐ Fairly positive

☐ Fairly negative

☐ Very negative

**Section 10 – Dental attendance**

The following question (59) is about how frequently you visit a dental professional.

59. **How often do you visit a dental professional (hygienist, dentist, other)?**

☐ At least every three months

☐ At least every six months

☐ At least once every year

☐ At least once every two years

☐ Less frequently than every two years

☐ Only when having trouble

☐ Never
